# Spinal cord hemorrhage in a patient with neurosarcoidosis on long-term corticosteroid therapy: case report

**DOI:** 10.1186/s12883-015-0373-6

**Published:** 2015-07-30

**Authors:** Benoit Pegat, Sophie Drapier, Xavier Morandi, Gilles Edan

**Affiliations:** Department of Neurology, Pontchaillou Hospital, Rennes University Hospital, Rennes, France; Department of Neurosurgery, Pontchaillou Hospital, Rennes University Hospital, Rennes, France

**Keywords:** Hematomyelia, Hemorrhage, Spinal cord, Neurosarcoidosis, Magnetic resonance imaging, Corticosteroid

## Abstract

**Background:**

Central nervous system bleeding is a rare complication of neurosarcoidosis: only 18 cases of spontaneous cerebral hematoma have been reported. We present the first recorded case of spinal cord hemorrhage in neurosarcoidosis.

**Case presentation:**

A 48-year-old Caucasian woman had relapsing neurosarcoidosis for 5 years, with inflammatory spinal and cerebral lesions. While on 20 mg corticosteroids, she experienced subacute paraparesia with right leg pain. A spine MRI revealed a low thoracic hematomyelia at the T10-T11 level. Despite high doses of corticosteroids, her condition continued to worsen. Surgical evacuation of the hematoma was performed 10 days after the onset of bleeding, and she partially recovered.

**Conclusion:**

This report highlights the possibility of spinal cord hemorrhage secondary to sarcoid vasculitis. The patient improved after surgical evacuation of the intramedullary hematoma. Immuno-modulating agents must be envisaged in severe neurosarcoidosis, to prevent complications.

## Background

Sarcoidosis is a multisystemic granulomatosis characterized by non-caseating granulomas [1]. In 5–7 % cases, it may involve the neurological system [2–4]. Spinal cord sarcoidosis occurs in about 6 % of all neurosarcoidosis cases [3, 5, 6]. Although several pathological studies have reported microvascular changes in neurosarcoidosis [7, 8], bleeding is rare. Only 18 cases of cerebral hemorrhage have been reported [9–12], and four of these concerned patients on corticosteroid therapy [10, 13–15]. We present the first recorded case of spinal cord hemorrhage in a 48-year-old Caucasian woman who had been treated with corticosteroids for 5 years for relapsing neurosarcoidosis.

## Case presentation

**Fig. 1:**
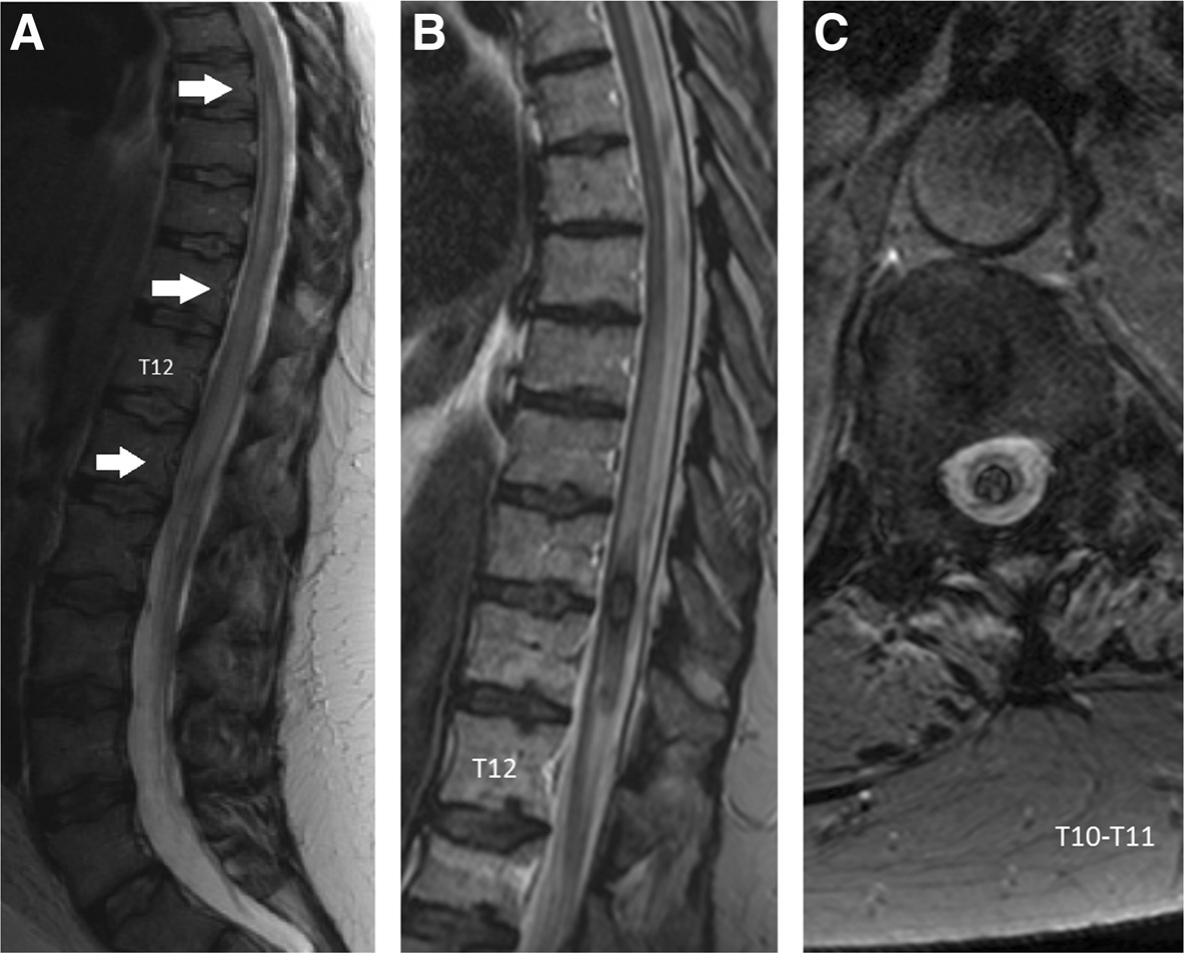
Spinal MRI images of the first inflammatory event (**a**) and the hematomyelia (**b** and **c**). **a**. Sagittal T2-weighted MRI showing a high-intensity signal and enlargement of the spinal cord extending from T8 to the L1-L2 level. **b**. Sagittal T2-weighted turbo spin-echo MRI showing a heterogeneous low-intensity signal at the T10-T11 level. **c**. Transversal T2*-weighted gradient-echo MRI at the T10–T11 level confirming the intramedullary low-intensity signal: it is an intramedullary hemorrhage (hematomyelia)

Sarcoidosis was histologically proven at 30 years, with pulmonary biopsy revealing non caseating granulomas when the patient presented with erythema nodosum and bilateral hilar adenopathy. Onset of neurosarcoidosis occurred 13 years later, characterized by cauda equina syndrome with a sensorimotor deficit in the right leg. Magnetic resonance imaging (MRI) revealed diffuse enlargement of the spinal cord, with high T2-weighted signal intensity from T8 to the L1-L2 level (Fig. 1a). There was no enhancement on gadolinium-enhanced T1-weighted images of the spine. Cerebral MRI was normal. Cerebrospinal fluid (CSF) tests revealed aseptic lymphocytic meningitis, with 540 cells/mm^3^ and a protein concentration of 375 mg/dL. Oligoclonal bands were absent, and the blood level of angiotensin-converting enzyme was normal. The patient’s condition improved after high doses of intravenous steroids, followed by oral corticosteroids (starting at 1 mg/kg/day, then tapering). CSF returned to normal after 4 months’ treatment. The leg dysesthesia persisted, as did bladder disturbances, treated by intermittent self-catheterization.

**Fig. 2:**
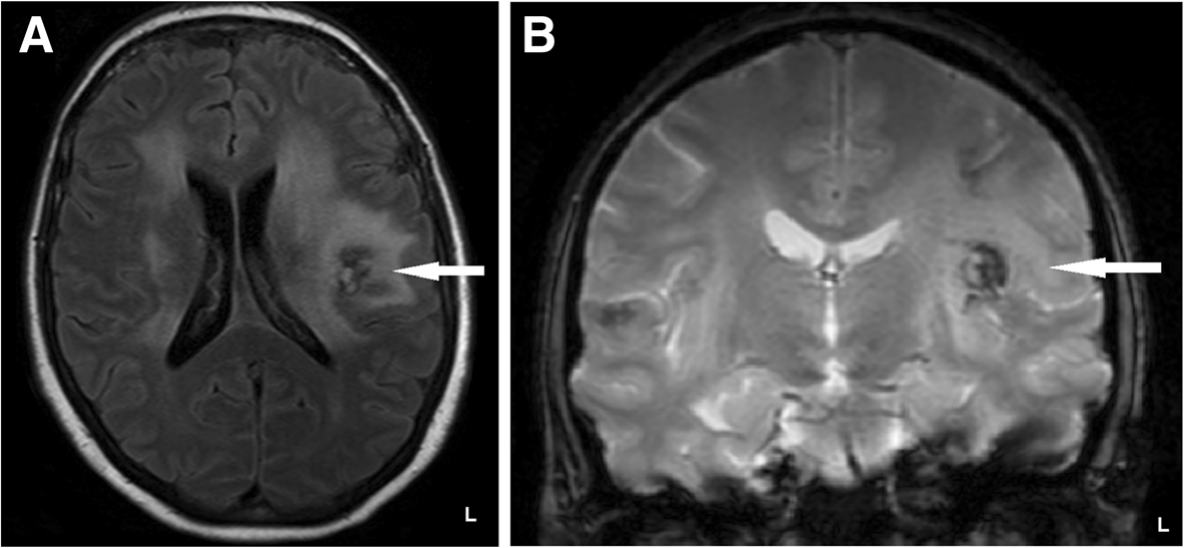
Cranial MRI images of the second inflammatory episode. **a**. Axial T2 fluid attenuation inversion recovery (FLAIR)-weighted MRI indicating large, high-intensity periventricular lesions, and a low-intensity left frontoparietal lesion. **b**. Coronal T2*-weighted gradient-echo MRI confirming the presence of a low-intensity lesion, suggestive of hemosiderin deposits, that had not been found on previous MRIs

Two years later, the patient had a relapse, while on steroids (4 mg/day), characterized by gait ataxia and cognitive impairment, inflammatory activity in the CSF (40 cells/mm^3^, protein concentration of 137 mg/dL) and multifocal lesions on a brain MRI (Fig. 2a-b). T2- fluid attenuation inversion recovery (FLAIR) and T2*-weighted gradient-echo imaging showed a low-intensity frontoparietal lesion identified as blood deposits. Steroids were increased to 1 mg/kg/day and she showed a marked improvement. Follow-up CSF analysis indicated fewer than 10 cells/mm^3^, and oligoclonal bands were found this time.

Three years later, while on corticosteroids (20 mg/day), the patient experienced paroxysmal right leg pain, acute worsening of gait difficulties (unable to walk alone owing to a major motor deficit in the right leg) and urinary retention. Sensory examination revealed a sensitive level at L3, with lower-limb hypoesthesia, and decreased pelvic sensation. Spinal MRI revealed a large heterogeneous intramedullary lesion at T10-T11, with a low-intensity T2*-weighted gradient-echo signal, diagnosed as hematomyelia (Fig. 1b-c). Part of the medullary cone was enhanced by gadolinium in a T1-weighted sequence. Coagulation blood tests and platelets were normal. Despite high doses of intravenous corticosteroids, she became paraplegic within 10 days, with a sensory deficit at the T12 level. A neurosurgical intervention was performed to evacuate the hematoma, without any additional complications. The neurosurgeon observed numerous abnormally enlarged blood vessels on the back of the spinal cord. Histological analysis of spinal cord tissue did not reveal any abnormal cells.

After yearlong intensive rehabilitation, the patient remained paraparetic, with a severe deficit in the right leg (3/5 on motor scale), but was nonetheless able to walk with a walking frame. Adding an immune-modulating agent was discussed but not done, because of a severe infectious complication of a pressure ulcer that required 6 weeks’ hospitalization in intensive care. Four years after the hematomyelia, the patient was on a daily dose of just 4 mg corticosteroids and remained relapse free, with no new lesions on follow-up MRI.

## Discussion

In the case described here, the diagnosis of neurosarcoidosis was *definite* according to Judson’s criteria [16] and *probable* according to Zajicek’s [17]. This first report highlights the possibility of spinal cord hemorrhage in patients with neurosarcoidosis, as suggested by Waubant and colleagues, who described hemosiderin deposits in spinal-cord sarcoidosis [18].

Common causes of atraumatic hematomyelia [19, 20] include vascular malformations (arteriovenous fistula, cavernoma, capillary telangiectasia, venous angioma) [21], neoplasms, Gowers’intrasyringal hemorrhage, spinal radiation, anticoagulation and bleeding disorders. None of these applied to our patient, suggesting that her hematomyelia was caused by bleeding of a spinal cord granuloma, given the proximity of the first inflammatory lesion (T8-L1) to the hemorrhage (T10-T11).

Despite the lack of histological confirmation of the source of bleeding, there is sufficient evidence from cerebral cases [9] to suggest that hemorrhage in neurosarcoidosis may be caused by vascular pathologies (venous, arteriolar, or micro-arterio-venous malformations) induced by the disease itself. Sarcoid granulomas in the central nervous system (CNS) follow perivascular spaces and penetrate brain parenchyma [22]. Post mortem examinations of intracranial hemorrhage in neurosarcoidosis have revealed perivascular inflammatory infiltrate and vessel wall destruction by granulomas [7, 9]. The enlarged blood vessels noticed by the neurosurgeon in our patient seem to support the hypothesis of neurosarcoid vasculitis. The frontoparietal hemorrhage that occurred during the second episode, 3 years before the hematomyelia (Fig. 2a-b), is also suggestive of the fragility of blood vessels in the CNS damaged by the neurosarcoid vasculitis.

Long-term corticosteroids also increase vascular fragility, owing to reduced collagen formation in vessel walls [23], but there was no evidence to suggest that steroids directly caused the bleeding here. Indeed, we decided to increase the corticosteroid dose, as adding an immuno-modulating agent to control the neurosarcoid vasculitis (and spare corticosteroids) was not possible because of an infectious complication. There have not been any randomized controlled trials of pharmacological treatments for neurosarcoidosis, but the algorithm developed by Nozaki and Judson, has proved useful for severe neurosarcoidosis [24].

Kreppel and colleagues’ meta-analysis [25] is the largest review of reports of spinal hematoma published before 1996. These authors reviewed 613 patients belonging to four etiological groups of spinal cord hemorrhage: intramedullary (hematomyelia), subarachnoidal, subdural and epidural. Hematomyelia was found in only 0.82 % of patients, possibly owing to the dearth of MRI-based diagnosis prior to 1996. More recently, Leep and colleagues [19] and Heldner and colleagues [20] reviewed the diagnosis, cause and treatment of hematomyelia. Hematomyelia can present as an acute, subacute, step-wise or chronic myelopathy [19]. As it did in our case, neurological deterioration can occur after the initial hemorrhage, owing to a secondary tissue response [19]. It is essential to monitor vital signs and neurological status in the intensive care unit, to prevent complications [20]. However, there have not been any clinical trials comparing different ways of managing hematomyelia [19, 20], notably the timing of hematoma evacuation. In our case, the decision to operate was made because of a progressive, rapid and inexorable neurological deterioration. There was a definite, but not total, clinical improvement following treatment for this rare and severe complication of neurosarcoidosis.

## Conclusion

This report presents the first case of intramedullary spinal cord hemorrhage complicating neurosarcoidosis, which improved following surgical evacuation. Neurosarcoid vasculitis may have favored this rare complication. Immuno-modulating agents could be envisaged as a means of preventing the complications of long-term corticosteroid therapy and vasculitis in severe CNS neurosarcoidosis.

### Consent

Written informed consent was obtained from the patient for publication of this Case report and any accompanying images. A copy of the written consent is available for review by the Editor of this journal.

## Abbreviations

CNS: Central nervous system
CSF: Cerebrospinal fluid
MRI: Magnetic resonance imaging
FLAIR: Fluid attenuation inversion recovery
